# *Sneathia amnii* and Maternal Chorioamnionitis and Stillbirth, Mozambique

**DOI:** 10.3201/eid2508.190526

**Published:** 2019-08

**Authors:** Pio Vitorino, Rosauro Varo, Paola Castillo, Juan Carlos Hurtado, Fabiola Fernandes, Ana Marta Valente, Rita Mabunda, Sibone Mocumbi, Joy M. Gary, Tiffany G. Jenkinson, Inacio Mandomando, Dianna M. Blau, Robert F. Breiman, Quique Bassat

**Affiliations:** Centro de Investigação em Saúde de Manhiça, Maputo, Mozambique (P. Vitorino, R. Varo, A.M. Valente, R. Mabunda, I. Mandomando, Q. Bassat);; ISGlobal Hospital Clinic–Universitat de Barcelona, Barcelona, Spain (R. Varo, P. Castillo, J.C. Hurtado, A.M. Valente, Q. Bassat);; Hospital Clínic, Barcelona (P. Castillo, J.C. Hurtado);; Hospital Central de Maputo, Maputo (F. Fernandes, S. Mocumbi);; Universidade Eduardo Mondlane, Maputo (F. Fernandes, S. Mocumbi);; Centers for Disease Control and Prevention, Atlanta, Georgia, USA (J.M. Gary, T.G. Jenkinson, D.M. Blau);; Emory Global Health Institute, Atlanta (R.F. Breiman);; Institució Catalana de Recerca i Estudis Avançats (ICREA), Barcelona (Q. Bassat);; Hospital Sant Joan de Déu, Barcelona (Q. Bassat);; Consorcio de Investigación Biomédica en Red de Epidemiología y Salud Pública (CIBERESP),; Madrid, Spain (Q. Bassat)

**Keywords:** *Sneathia amnii*, gram-negative infection, chorioamnionitis, stillbirth, bacteria, Mozambique

## Abstract

We report a case of *Sneathia amnii* as the causative agent of maternal chorioamnionitis and congenital pneumonia resulting in a late fetal death in Mozambique, with strong supportive postmortem molecular and histopathologic confirmation. This rare, fastidious gram-negative coccobacillus has been reported to infrequently cause abortions, stillbirths, and neonatal infections.

*Sneathia amnii,* formerly designated *Leptotrichia amnionii,* is a rare, fastidious, gram-negative coccobacillus, first described in the amniotic fluid of a woman with a fetal demise (*1*). The inherent difficulties in conventionally culturing this pathogen led to its initial identification through analyzing the 16S rRNA gene; its genome was recently sequenced (*1*,*2*). *S. amnii* is an opportunistic agent of the female urogenital tract (*3*,*4*) associated with cases of spontaneous abortion (miscarriage) and neonatal meningitis (*1*,*5*,*6*). We describe a perinatal case of *S. amnii* infection in a mother–fetus dyad, which we documented and investigated with the minimally invasive tissue sampling (MITS) postmortem procedure (*7*).

An otherwise healthy multigravida 37-year-old woman, at an estimated gestational age of 39 weeks, was admitted to Manhiça District Hospital, southern Mozambique, in labor. During pregnancy, she had attended 2 antenatal consultations and received the standard of care for pregnant women in Mozambique; mild anemia was treated with ferrous sulfate and folic acid supplements. Serologic tests for syphilis and HIV were both negative. Upon arrival at the hospital, the mother was afebrile and hemodynamically stable; she had a fully effaced uterine cervix, thin and elastic, 2 cm dilation; intact amniotic membranes; and cephalic fetal presentation with heartbeat present. Physical examination did not provide additional information. Labor progressed with spontaneous rupture of membranes. No additional documentation of the fetal heartbeat was available before delivery. Two hours after arrival, a fresh stillborn female weighing 3.5 kg was born by spontaneous vaginal delivery. Size was normal, and no macroscopic congenital abnormalities were observed. The mother was discharged next day without complications.

As part of Mozambique’s Child Health and Mortality Prevention Surveillance (CHAMPS), after obtaining written, informed consent, we conducted MITS by biopsy needle of tissues and body fluids, in addition to placenta, to ascertain the cause of the stillbirth (*7*). Samples are subject to thorough histopathologic, molecular, and microbiological investigation, including universal screening for HIV-1, *Mycobacterium tuberculosis*, and malaria parasites. We performed conventional microbiological cultures of blood and cerebrospinal fluid (CSF); we inoculated ≈3 mL of blood into aerobic blood culture bottle (BACTEC system; Becton Dickinson, https://www.bd.com) and cultured CSF samples into blood, chocolate, and MacConkey agar plates. We performed multipathogen molecular screening using TaqMan Array Card (Applied Biosystems, https://www.thermofisher.com) in whole blood, CSF, lung, and rectal swab samples (*8*). We prepared and examined tissue samples using conventional pathologic methods and targeted immunohistochemical staining (*9*).

We isolated no microorganisms in CSF or blood, nor did we detect a likely pathogen in any of the unfixed postmortem tissues. At CHAMPS reference pathology laboratories, examination of tissue samples showed similar morphological findings in placental and miscellaneous tissues that suggested infection, including an acute inflammatory infiltrate in the lungs compatible with bronchopneumonia. We also found moderate numbers of aspirated squames and increased alveolar macrophages, indicating intrauterine fetal distress and associated aspiration of amniotic fluid. No aspirated meconium was apparent. Gram stain revealed gram-negative coccobacilli in alveoli and adjacent bronchioles. We conducted a cross-reactive immunohistochemical assay targeting multiple bacteria in the lung samples using paneubacteria and gram type–specific PCR assays targeting the 16S rRNA gene; we identified *S. amnii* by sequence analysis of positive amplicons (Figure, panels A–C). We observed no remarkable histopathologic findings in the liver or brain, and the cross-reactive polybacterial immunohistochemical assay was negative in brain tissue. Placental tissue and umbilical cord showed an acute chorioamnionitis with maternal response (inflammation in the membranes, stage 2) and fetal response (inflammation in the umbilical cord, stage 2) showing umbilical arteritis with rare gram-negative coccobacilli. There was no immunohistochemical evidence of bacteria in this tissue (Figure, panel D). We obtained an amplicon from placental tissue by paneubacteria PCR; however, we could not confirm the presence of *S. amnii* sequences. 

**Figure:**
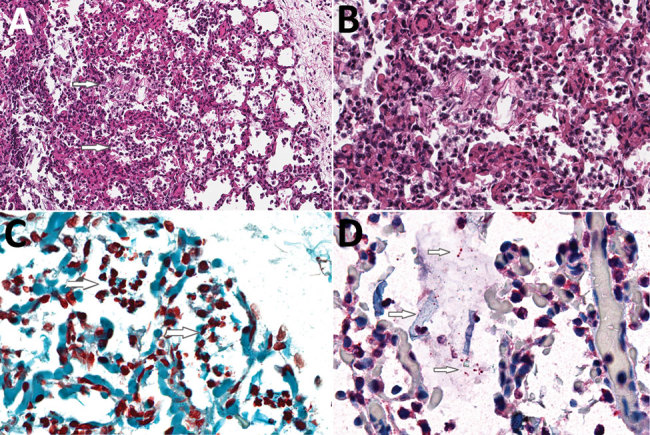
Histologic evidence of amniotic fluid aspiration, bronchopneumonia, and intraalveolar gram-negative coccobacilli in the lung of a stillborn infant, Mozambique. A) Hematoxylin and eosin stain of lung tissue showing acute inflammation within alveoli (bronchopneumonia, upper arrow) and moderate numbers of aspirated squames (lower arrow), consistent with intrauterine fetal distress and associated aspiration of amniotic fluid. Original magnification ×20. B) Higher magnification of panel A tissue showing acute inflammation within alveoli (bronchopneumonia) and a clump of aspirated squames. Original magnification ×40. C) Gram stain of lung showing multiple small, gram-negative coccobacilli mixed with acute inflammation within alveoli (arrows indicate regions with bacteria). Original magnification ×63. D) Polybacterial immunohistochemical assay of lung tissue targeting multiple bacteria highlights the coccobacilli within alveoli (top and bottom arrows). Aspirated squames are also present (middle arrow). Original magnification ×63.

CHAMPS procedures include the review of all clinical, microbiological, molecular, and histopathological data, along with the verbal autopsy, by a multidisciplinary panel of local experts (D.M. Blau et al., unpub. data). The panel concluded that the immediate cause of this stillbirth could be attributed to a congenital pneumonia, caused by *S. amnii*, that could have originated in the mother’s placenta; we determined that chorioamnionitis was the main maternal condition associated with the child’s death. The presence of *Sneathia* sp. bacteria in amniotic fluid can lead to inflammation and histologic chorioamnionitis, amnionitis, or both (*10*). 

*S. amnii* has been identified in different settings as a pathologic agent in women and children (*1*,*3*–*6*). In this case in a rural setting in Africa, *S. amnii* was the causative agent in a stillbirth with congenital pneumonia, a diagnosis supported by strong postmortem molecular and histopathologic confirmation. As CHAMPS evaluation continues in Mozambique, as well as at sites in 6 additional countries in sub-Saharan Africa and south Asia, we expect the importance of this pathogen to become clearer.
